# Experimentally determined trace element partition coefficients between hibonite, melilite, spinel, and silicate melts

**DOI:** 10.1016/j.dib.2018.10.100

**Published:** 2018-10-27

**Authors:** D. Loroch, S. Klemme, J. Berndt, A. Rohrbach

**Affiliations:** Institute for Mineralogy, University of Münster, Corrensstrasse 24, 48149 Münster, Germany

## Abstract

This article provides new data on mineral/melt partitioning in systems relevant to the evolution of chondrites, Calcium Aluminum-Rich Inclusions (CAI) in chondrites and related meteorites. The data set includes experimentally determined mineral/melt partition coefficients between hibonite (CaAl_12_O_19_), melilite (Ca_2_(Al,Mg)_2_SiO_7_), spinel (MgAl_2_O_4_) and silicate melts for a wide range of trace elements: Sc, Ti, V, Cr, Co, Ni, Cu, Zn, Ga, Ge, Rb, Sr, Y, Zr, Nb, Rh, Cs, Ba, La, Ce, r, Nd, Sm, Eu, Gd, Tb, Dy, Ho, Er, Tm, Yb, Lu, Hf, Ta, W, Pb, Th and U. The experiments were performed at high temperatures (1350 °C < *T* < 1550 °C) and ambient pressure. The experimental run products were analyzed using electron microprobe (EMPA) and laser ablation inductively coupled plasma mass spectrometry (LA-ICP-MS). The partition coefficients for 38 trace elements were calculated from the LA-ICP-MS data.

**Specifications table**

**Table:** 

Subject area	*Earth Sciences*
More specific subject area	Experimental petrology, Geochemistry, Planetology, Planetary sciences
Type of data	*Table, figure*
How data was acquired	*High-temperature furnace: Gero GmbH, Germany (University of Münster)*
	*Scanning electron microscope (SEM) JEOL JSM-6610 LV in high vacuum mode equipped with EDX system (University of Münster)*
	*Electron microprobe analysis (EMPA): JEOL JXA-8530F Hyperprobe equipped with a field emission gun (University of Münster)*
	*Laser ablation inductively coupled plasma mass spectrometry (LA-ICP-MS): Thermo element sector field – ICP-MS with Photon Machines Analyte G2 laser ablation system (University of Münster)*
Data format	*Major element data of minerals and quenched melts: data in .xlsx format*
	*Trace element data of minerals and quenched melts: data in .xlsx format*
	*Mineral/melt trace element partition coefficients: data in .xlsx format*
	*Mineral/mineral trace element partition coefficients: data in .xlsx format*
Experimental features	*High temperature experiments were run at high temperatures to equilibrate hibonite, melilite, and spinel, with silicate melts. The experimental run products were mounted in epoxy resins and polished using a variety of diamond pastes. The mounts were carbon coated, and major elements were analyzed using EMPA techniques. Subsequently, trace element concentrations of minerals and glasses within the samples were determined using LA-ICP-MS techniques.*
Data accessibility	Supplementary materials

**Value of the data**

• The new trace element partition coefficients supplement the existing database of mineral/melt partition coefficients of minerals that are frequently found in Ca- and Al-rich inclusions in chondritic meteorites.
• The new trace element partition coefficients between hibonite, melilite and spinel and silicate melts may be used to test whether these minerals crystallized from or equilibrated with a silicate melt or whether they condensed from a vapor phase.
• This partition coefficient data set is based on experiments under oxidizing conditions, since preliminary experiments under reducing conditions, which would have been more relevant to solar nebula processes, resulted in crystals which were too small to be analyzed.
• Our mineral/mineral partition coefficients may be used to test whether hibonite, melilite and spinel are in thermodynamic equilibrium or not.

## 1 Data

In this article, we report new experimentally determined trace element partition coefficients between hibonite (CaAl_12_O_19_), melilite (Ca_2_(Al,Mg)_2_SiO_7_), spinel (MgAl_2_O_4_), and silicate melts at high temperatures (Tables 3 and 4). Data were generated using high temperature experiments, which were characterized using electron microprobe and LA-ICP-MS methods (Table 1, Table 4, Table 5, Table 6).

**Table 1:** Major element concentrations of minerals and quenched silicate melts determined by EMPA. All values are given in wt%.

Sample	MgO		Al_2_O_3_		SiO_2_		CaO		TiO_2_	
	wt%	S.D.	wt%	S.D.	wt%	S.D.	wt%	S.D.	wt%	S.D.
	**Hibonite**									
H1-Ti2-R3	1.50	±0.14	86.8	±0.1	0.86	±0.14	8.33	±0.10	1.77	±0.21
H1-Ti5-R4	2.01	±0.13	85.1	±0.7	0.64	±0.13	8.36	±0.08	3.16	±0.41
H1-Ti5-R5	2.02	±0.47	84.9	±1.6	0.70	±0.17	8.39	±0.06	3.19	±0.97
H2-Ti2-R2	1.87	±0.06	85.7	±0.3	1.22	±0.12	8.27	±0.09	1.84	±0.05
H2-Ti2-R3	1.96	±0.28	85.5	±2.0	1.12	±0.25	8.29	±0.14	2.23	±0.62
H2-Ti5-R4	2.41	±0.07	83.8	±0.3	0.90	±0.19	8.30	±0.09	3.61	±0.16
H2-Ti5-R5	2.53	±0.18	83.6	±0.8	0.91	±0.15	8.34	±0.07	3.86	±0.57
H3-Ti5-R4	2.37	±0.06	84.8	±0.4	0.81	±0.11	8.27	±0.09	3.57	±0.10
H3-Ti5-R5	2.79	±0.11	82.7	±0.9	0.96	±0.39	8.39	±0.10	4.37	±0.41
	**Melilite**									
H1-Ti2-R3	0.175	±0.031	35.7	±0.4	22.1	±0.2	40.7	±0.2	0.046	±0.042
H3-R8	0.318	±0.030	35.1	±1.4	21.6	±0.3	40.9	±0.2	–	–
	**Spinel**									
H2-R8	25.3	±0.2	72.1	±0.1	0.026	±0.012	0.021	±0.004	–	–
H3-R8	19.7	±1.1	78.3	±1.3	0.034	±0.043	0.026	±0.014	–	–
Mel3-R9	28.0	±0.2	70.9	±0.4	0.022	±0.036	0.014	±0.004	0.057	±0.047
Mel3-R11	28.0	±0.2	70.5	±0.9	0.037	±0.024	0.016	±0.007	0.081	±0.028
Mel3-R12	27.9	±0.2	70.9	±0.2	0.024	±0.032	0.011	±0.009	0.059	±0.052
	**Silicate Melt**									
H1-Ti2-R3	0.79	±0.13	34.0	±0.5	32.2	±0.9	28.6	±0.3	2.05	±0.21
H1-Ti5-R4	0.71	±0.02	35.2	±0.5	28.8	±2.0	27.2	±0.3	4.97	±0.40
H1-Ti5-R5	0.66	±0.09	31.8	±0.6	31.6	±2.2	29.1	±0.5	4.60	±0.40
H2-Ti2-R2	1.41	±0.19	36.0	±0.5	32.7	±1.1	24.5	±0.1	1.86	±0.31
H2-Ti2-R3	1.38	±0.21	33.2	±0.2	34.9	±1.3	25.6	±0.4	1.92	±0.3
H2-Ti5-R4	1.81	±0.22	34.9	±0.3	32.9	±1.5	23.9	±0.3	4.29	±0.38
H2-Ti5-R5	1.59	±0.16	31.3	±0.4	34.7	±1.0	25.3	±0.4	4.25	±0.24
H2-R8	2.38	±0.17	36.2	±0.7	31.3	±0.8	28.1	±0.3	–	–
H3-Ti5-R4	2.07	±0.12	35.8	±0.8	28.2	±1.2	26.8	±0.6	4.23	±0.33
H3-Ti5-R5	1.90	±0.18	31.2	±0.8	36.2	±1.8	28.8	±0.1	4.42	±0.24
H3-R8	1.69	±0.10	37.4	±1.3	31.6	±2.7	27.2	±0.4	–	–
Mel3-R9	6.04	±0.12	19.1	±2.1	36.1	±4.1	36.3	±3.0	1.02	±0.15
Mel3-R11	5.33	±0.50	19.1	±1.4	38.4	±1.1	31.5	±3.9	2.16	±0.22
Mel3-R12	6.13	±0.67	18.9	±4.3	38.5	±5.7	32.2	±7.1	2.18	±0.91

**Table 2:** Trace element concentrations of minerals and quenched silicate melts determined with LA-ICP-MS. All values are given in µg/g.

	**Hibonite**													
	H1-Ti2-R3		H1-Ti5-R4		H1-Ti5-R5		H2-Ti2-R2		H2-Ti2-R3		H2-Ti5-R4		H2-Ti5-R5	
	µg/g	S.D.	µg/g	S.D.	µg/g	S.D.	µg/g	S.D.	µg/g	S.D.	µg/g	S.D.	µg/g	S.D.
Mg	13367	± 2518	15263	± 2195	14105	± 3235	14245	± 2822	17157	± 3259	18699	± 2799	20315	± 5382
Si	7058	± 1008	3778	± 668	4712	± 780	5629	± 863	9282	± 1272	5229	± 841	4912	± 758
Ca	59563	± 2104	59749	± 2165	59978	± 2172	59077	± 2166	59234	± 2095	59349	± 2167	59577	± 2119
Sc	30.6	± 2.0	28.2	± 2.1	28.3	± 2.2	28.7	± 2.0	28.3	± 1.9	24.6	± 1.9	25.5	± 1.9
Ti	11302	± 1412	21355	± 1797	19485	± 2546	12899	± 1736	14804	± 1124	24027	± 2099	28123	± 3082
V	6.26	± 0.57	3.46	± 0.47	3.44	± 0.53	5.70	± 0.62	5.98	± 0.56	4.87	± 0.59	3.82	± 0.49
Cr	29.1	± 8.3	32.1	± 9.8	25.0	± 9.1	23.9	± 9.0	27.9	± 7.6	20.7	± 9.7	23.8	± 7.6
Co	185	± 9	280	± 16	270	± 21	123	± 6	132	± 7	199	± 12	226	± 13
Ni	327	± 85	353	± 51	320	± 76	137	± 44	201	± 55	249	± 41	369	± 126
Cu	11.0	± 1.1	11.8	± 1.2	8.67	± 1.09	12.2	± 1.2	11.5	± 1.0	9.48	± 1.16	7.98	± 0.91
Zn	13.7	± 3.4	14.7	± 4.8	14.7	± 4.1	12.4	± 3.5	12.9	± 3.3	14.2	± 4.6	12.1	± 3.3
Ga	247	± 13	180	± 9	175	± 11	200	± 11	258	± 14	233	± 11	229	± 15
Ge	7.31	± 2.42	8.23	± 2.84	7.00	± 2.65	9.32	± 2.68	8.04	± 2.25	10.7	± 2.9	9.32	± 2.32
Rb	b.d.l		b.d.l		b.d.l		b.d.l		b.d.l		0.93	± 0.31	0.57	± 0.18
Sr	92.5	± 4.0	94.4	± 4.4	94.7	± 5.5	91.8	± 4.2	93.0	± 4.1	94.8	± 4.5	98.8	± 4.9
Y	36.7	± 2.1	23.4	± 1.4	25.5	± 1.9	53.8	± 3.2	49.0	± 2.8	32.2	± 1.9	30.1	± 2.2
Zr	27.6	± 1.7	21.8	± 1.4	21.1	± 1.5	30.1	± 2.0	28.9	± 1.8	18.5	± 1.2	21.0	± 1.7
Nb	9.63	± 0.59	8.17	± 0.55	8.10	± 0.58	20.3	± 1.2	20.3	± 1.1	10.2	± 0.6	10.8	± 0.7
Rh	7.14	± 0.74	4.52	± 0.55	4.91	± 0.94	4.25	± 0.54	6.29	± 0.69	7.30	± 0.88	8.87	± 1.40
Cs	b.d.l		0.43	± 0.18	0.41	± 0.15	b.d.l		0.33	± 0.12	b.d.l		b.d.l	
Ba	3.57	± 0.75	2.76	± 0.78	3.33	± 0.84	2.84	± 0.88	3.09	± 0.75	2.94	± 0.77	3.40	± 0.86
La	378	± 16	291	± 11	286	± 14	589	± 26	484	± 20	446	± 18	426	± 23
Ce	454	± 19	354	± 15	346	± 19	608	± 27	496	± 21	447	± 19	450	± 23
Pr	372	± 15	281	± 13	277	± 18	421	± 18	354	± 15	306	± 15	303	± 15
Nd	319	± 16	235	± 12	232	± 16	428	± 23	366	± 19	305	± 15	297	± 20
Sm	247	± 14	180	± 10	178	± 13	316	± 20	280	± 16	223	± 12	218	± 18
Eu	143	± 7	102	± 5	102	± 7	183	± 9	160	± 8	127	± 6	121	± 8
Gd	142	± 8	104	± 7	106	± 11	217	± 13	186	± 11	142	± 10	140	± 11
Tb	95.1	± 5.1	67.4	± 3.4	70.3	± 5.1	139	± 8	120	± 6	87.9	± 4.6	83.6	± 6.1
Dy	46.6	± 3.3	31.5	± 2.1	33.7	± 3.1	64.8	± 4.9	55.8	± 4.0	39.6	± 2.7	36.4	± 3.7
Ho	42.4	± 2.6	28.7	± 1.6	30.8	± 2.4	58.9	± 3.9	52.2	± 3.3	35.9	± 2.0	32.4	± 2.9
Er	15.9	± 1.3	11.6	± 0.9	11.6	± 1.1	26.0	± 2.1	22.4	± 1.7	14.3	± 1.0	13.8	± 1.5
Tm	9.56	± 0.65	5.88	± 0.38	6.79	± 0.55	15.6	± 1.1	13.7	± 0.9	8.38	± 0.52	7.56	± 0.72
Yb	11.3	± 1.0	7.44	± 0.75	8.00	± 0.94	15.3	± 1.4	13.9	± 1.2	8.49	± 0.84	7.70	± 0.89
Lu	7.52	± 0.49	4.43	± 0.31	4.91	± 0.42	11.8	± 0.8	10.5	± 0.7	6.05	± 0.40	5.62	± 0.50
Hf	39.6	± 2.5	34.2	± 2.2	33.7	± 3.1	62.1	± 4.1	55.8	± 3.5	40.4	± 2.6	43.8	± 3.7
Ta	40.1	± 2.2	35.7	± 1.9	36.8	± 2.8	84.6	± 4.9	81.9	± 4.4	45.9	± 2.5	52.0	± 3.9
W	0.09	± 0.06	0.10	± 0.07	0.20	± 0.10	0.28	± 0.13	0.19	± 0.09	0.13	± 0.10	0.29	± 0.11
Pb	b.d.l		b.d.l		0.27	± 0.11	b.d.l		b.d.l		b.d.l		b.d.l	
Th	131	± 7	71.5	± 3.5	72.3	± 5.0	191	± 11	162	± 9	110	± 5	106	± 8
U	b.d.l		0.01	± 0.01	b.d.l		0.02	± 0.01	0.01	± 0.01	0.01	± 0.01	0.01	± 0.01


	**Hibonite**				**Melilite**				**Spinel**					
	H3-Ti5-R4		H3-Ti5-R5		H1-Ti2-R3		H3-R8		H2-R8		H3-R8		Mel3-R9	
	µg/g	S.D.	µg/g	S.D.	µg/g	S.D.	µg/g	S.D.	µg/g	S.D.	µg/g	S.D.	µg/g	S.D.
Mg	16888	± 2734	20955	± 5971	55758	±10561	2525	± 242	166434	±36418	124887	± 9030	176044	±17334
Si	17111	± 2303	7555	± 1118	110371	±14987	104108	±13955	b.d.l		4197	± 529	b.d.l	
Ca	59120	± 2034	59935	± 2154	290740	±12078	292070	± 9299	1627	± 681	1872	± 554	1044	± 348
Sc	44.5	± 2.7	52.8	± 3.9	189	± 14	3.23	± 0.36	15.4	± 2.2	33.6	± 2.1	16.8	± 1.3
Ti	21275	± 1994	29299	± 3489	57273	± 4394	3.78	± 1.35	b.d.l		16.2	± 4.8	372	± 34
V	16.3	± 1.2	7.11	± 0.74	109	± 9	0.39	± 0.09	1.04	± 0.50	4.79	± 0.39	0.62	± 0.24
Cr	24.9	± 6.3	23.7	± 8.3	b.d.l		b.d.l		b.d.l		18.5	± 9.8	25.5	± 6.7
Co	142	± 9	196	± 11	686	± 38	18.2	± 1.1	3577	± 467	1409	± 94	413	± 39
Ni	144	± 25	207	± 78	1206	± 354	17.5	± 5.9	12392	± 5282	3732	± 515	1098	± 216
Cu	10.4	± 1.0	5.93	± 0.87	93.8	± 9.9	2.42	± 0.32	122	± 17	73.3	± 5.3	7.87	± 1.01
Zn	8.41	± 2.71	8.75	± 3.25	73.0	± 25.4	1.45	± 0.70	137	± 48	37.1	± 9.2	9.32	± 3.82
Ga	236	± 12	244	± 17	815	± 47	112	± 6	577	± 69	258	± 12	373	± 21
Ge	6.28	± 1.74	8.93	± 2.46	b.d.l		2.73	± 0.92	13.3	± 5.5	b.d.l		5.03	± 2.20
Rb	b.d.l		b.d.l		b.d.l		0.14	± 0.06	0.82	± 0.34	b.d.l		0.27	± 0.15
Sr	86.3	± 4.1	107	± 5	422	± 21	110	± 5	b.d.l		5.02	± 0.41	b.d.l	
Y	28.7	± 1.7	27.0	± 2.2	189	± 12	93.2	± 4.1	b.d.l		4.59	± 0.18	0.20	± 0.08
Zr	23.0	± 1.4	34.0	± 2.7	169	± 13	b.d.l		b.d.l		6.04	± 1.75	b.d.l	
Nb	12.6	± 0.7	21.4	± 1.4	117	± 8	0.03	± 0.02	0.14	± 0.07	1.18	± 0.09	0.03	± 0.03
Rh	6.31	± 0.81	8.78	± 1.51	45.2	± 5.4	0.05	± 0.02	b.d.l		9.49	± 0.81	14.4	± 1.5
Cs	b.d.l		b.d.l		1.53	± 1.00	0.08	± 0.03	b.d.l		b.d.l		b.d.l	
Ba	9.20	± 1.06	4.85	± 1.04	61.9	± 10.5	1.15	± 0.23	1.60	± 0.79	3.65	± 0.06	0.88	± 0.35
La	319	± 13	336	± 19	1176	± 53	51.2	± 1.7	b.d.l		0.11	± 0.01	b.d.l	
Ce	359	± 16	379	± 20	1522	± 69	74.1	± 2.6	0.03	± 0.06	0.19	± 0.01	0.02	± 0.03
Pr	244	± 12	269	± 14	1258	± 57	76.6	± 2.7	b.d.l		0.08	± 0.01	0.03	± 0.02
Nd	215	± 11	237	± 17	1082	± 63	91.1	± 3.9	0.15	± 0.24	0.13	± 0.02	0.13	± 0.09
Sm	158	± 9	182	± 16	930	± 61	129	± 6	0.50	± 0.30	0.24	± 0.05	0.05	± 0.08
Eu	87.1	± 4.5	99.8	± 6.9	555	± 30	93.0	± 3.6	b.d.l		0.07	± 0.01	0.03	± 0.03
Gd	87.6	± 6.4	104	± 9	525	± 38	124	± 6	b.d.l		0.25	± 0.03	0.09	± 0.14
Tb	56.4	± 3.1	64.5	± 5.1	392	± 23	107	± 4	0.06	± 0.04	0.12	± 0.01	0.03	± 0.02
Dy	31.8	± 2.2	34.7	± 3.8	189	± 17	78.2	± 3.4	0.10	± 0.19	0.12	± 0.02	0.04	± 0.04
Ho	31.6	± 1.8	33.8	± 3.3	222	± 15	93.8	± 3.9	0.05	± 0.04	0.10	± 0.01	0.05	± 0.02
Er	13.2	± 0.9	12.7	± 1.5	101	± 10	44.8	± 2.0	0.14	± 0.11	0.10	± 0.01	0.08	± 0.04
Tm	9.54	± 0.57	8.28	± 0.84	77.5	± 5.9	36.5	± 1.4	0.11	± 0.05	0.06	± 0.01	0.08	± 0.03
Yb	14.0	± 1.1	10.7	± 1.2	109	± 11	54.7	± 2.3	0.17	± 0.17	0.14	± 0.02	0.16	± 0.08
Lu	12.3	± 0.7	8.70	± 0.80	101	± 7	46.7	± 1.9	0.14	± 0.06	0.12	± 0.01	0.15	± 0.03
Hf	32.4	± 2.2	53.4	± 4.8	200	± 15	0.05	± 0.02	0.29	± 0.17	0.14	± 0.02	0.16	± 0.07
Ta	35.9	± 2.0	70.5	± 5.6	313	± 18	0.02	± 0.01	0.07	± 0.04	0.20	± 0.01	0.03	± 0.02
W	1.62	± 0.23	1.57	± 0.29	6.59	± 1.95	0.03	± 0.03	0.27	± 0.14	0.31	± 0.02	0.07	± 0.05
Pb	b.d.l		b.d.l		b.d.l		b.d.l		b.d.l		b.d.l		b.d.l	
Th	68.4	± 3.5	74.2	± 6.4	490	± 29	9.05	± 0.35	0.06	± 0.03	0.95	± 0.07	0.01	± 0.01
U	0.05	± 0.02	0.03	± 0.01	0.13	± 0.10	0.00	± 0.00	b.d.l		0.01	± 0.01	b.d.l	


	**Spinel**				**Silicate Melt**									
	Mel3-R11		Mel3-R12		H1-Ti2-R3		H1-Ti5-R4		H1-Ti5-R5		H2-Ti2-R2		H2-Ti2-R3	
	µg/g	S.D.	µg/g	S.D.	µg/g	S.D.	µg/g	S.D.	µg/g	S.D.	µg/g	S.D.	µg/g	S.D.
Mg	186262	±33494	181809	±31474	7171	± 1349	5695	± 822	4735	± 1125	15407	± 3107	15370	± 2944
Si	1293	± 336	1010	± 304	157158	±20531	140628	±18404	151388	±21120	170836	±22512	182917	±23969
Ca	b.d.l		1190	± 339	204571	± 6514	194023	±6185	210641	± 6714	174887	± 5580	182874	± 5835
Sc	13.1	± 2.2	12.4	± 1.7	169	± 9	146	± 8	163	± 11	147	± 8	156	± 9
Ti	369	± 57	333	± 48	12438	± 922	31610	± 2649	29161	± 3938	11937	± 965	12212	± 930
V	0.54	± 0.24	0.58	± 0.23	192	± 11	169	± 10	183	± 15	129	± 8	121	± 7
Cr	34.1	± 10.1	46.5	± 10.5	b.d.l		6.12	± 2.33	b.d.l		b.d.l		12.3	± 2.5
Co	425	± 70	425	± 51	113	± 5	117	± 7	117	± 9	139	± 7	117	± 6
Ni	1014	± 251	1030	± 223	284	± 71	185	± 25	194	± 47	222	± 59	261	± 66
Cu	73.2	± 10.6	26.5	± 3.0	71.1	± 4.2	65.7	± 4.0	48.1	± 3.9	176	± 11	97.5	± 5.8
Zn	23.5	± 5.0	15.7	± 4.0	4.08	± 0.97	4.16	± 1.21	3.40	± 1.14	5.61	± 1.16	5.27	± 1.15
Ga	369	± 60	413	± 49	96.3	± 4.9	99.5	± 4.6	89.5	± 5.4	121	± 6	106	± 5
Ge	b.d.l		b.d.l		2.44	± 0.66	2.94	± 0.73	2.27	± 0.73	2.48	± 0.78	2.07	± 0.75
Rb	0.44	± 0.18	b.d.l		0.40	± 0.08	0.52	± 0.10	0.44	± 0.09	1.23	± 0.13	0.70	± 0.10
Sr	0.72	± 0.38	b.d.l		188	± 8	171	± 7	184	± 10	178	± 7	183	± 7
Y	0.10	± 0.06	0.07	± 0.05	163	± 9	142	± 7	164	± 12	149	± 8	159	± 9
Zr	b.d.l		0.09	± 0.07	138	± 7	118	± 5	132	± 8	107	± 6	116	± 6
Nb	b.d.l		0.03	± 0.02	110	± 5	98.7	± 4.3	108	± 6	100	± 5	107	± 5
Rh	32.9	± 6.4	36.1	± 6.0	0.37	± 0.06	0.33	± 0.05	0.25	± 0.06	b.d.l		0.18	± 0.04
Cs	b.d.l		0.24	± 0.12	b.d.l		0.11	± 0.05	b.d.l		b.d.l		b.d.l	
Ba	b.d.l		b.d.l		116	± 6	101	± 6	115	± 10	114	± 6	120	± 6
La	b.d.l		b.d.l		50.3	± 2.0	67.9	± 2.5	55.3	± 2.8	83.3	± 3.5	57.4	± 2.3
Ce	0.03	± 0.03	0.04	± 0.02	101	± 4	122	± 5	113	± 6	117	± 5	85.3	± 3.5
Pr	0.06	± 0.03	0.02	± 0.02	79.6	± 3.2	95.6	± 4.3	86.0	± 5.6	79.5	± 3.3	62.1	± 2.6
Nd	0.10	± 0.09	0.09	± 0.08	80.4	± 4.0	93.3	± 4.4	86.1	± 5.8	92.9	± 4.9	77.1	± 3.9
Sm	0.16	± 0.11	0.09	± 0.07	126	± 7	132	± 6	133	± 10	124	± 7	115	± 7
Eu	b.d.l		0.04	± 0.05	106	± 5	108	± 5	110	± 8	99.6	± 4.9	96.5	± 4.6
Gd	b.d.l		b.d.l		127	± 7	124	± 8	130	± 13	137	± 8	137	± 8
Tb	0.01	± 0.02	0.04	± 0.02	146	± 8	134	± 6	148	± 11	139	± 8	144	± 8
Dy	0.08	± 0.06	0.06	± 0.05	108	± 7	98.5	± 5.4	110	± 10	96.0	± 6.8	101	± 7
Ho	0.05	± 0.02	0.02	± 0.01	151	± 9	134	± 7	152	± 12	130	± 9	137	± 8
Er	0.07	± 0.05	0.10	± 0.05	89.8	± 5.9	79.4	± 3.9	89.9	± 6.8	86.0	± 6.2	90.8	± 6.2
Tm	0.05	± 0.03	0.10	± 0.03	89.2	± 5.3	78.8	± 3.7	88.6	± 6.3	83.6	± 5.4	89.8	± 5.5
Yb	0.07	± 0.09	0.14	± 0.11	164	± 9	144	± 8	161	± 14	129	± 8	141	± 8
Lu	0.10	± 0.04	0.06	± 0.03	142	± 8	124	± 6	140	± 10	134	± 8	146	± 8
Hf	0.04	± 0.06	0.09	± 0.06	80.5	± 4.6	72.2	± 4.0	80.1	± 7.1	94.1	± 5.7	98.3	± 5.7
Ta	0.01	± 0.03	0.01	± 0.02	114	± 6	103	± 5	113	± 8	114	± 6	118	± 6
W	0.03	± 0.05	b.d.l		6.94	± 0.50	13.6	± 0.8	22.6	± 1.9	5.26	± 0.42	4.48	± 0.37
Pb	b.d.l		0.27	± 0.08	0.08	± 0.03	0.09	± 0.03	b.d.l		0.15	± 0.04	0.09	± 0.03
Th	b.d.l		0.01	± 0.01	84.2	± 4.6	83.6	± 3.8	88.7	± 6.2	83.1	± 4.9	80.4	± 4.5
U	0.01	± 0.01	b.d.l		0.37	± 0.03	0.53	± 0.04	0.42	± 0.04	0.55	± 0.05	0.27	± 0.03


	**Silicate Melt**													
	H3-Ti5-R4		H3-Ti5-R5		H3-R8		Mel3-R9		Mel3-R11		Mel3-R12			
	µg/g	S.D.	µg/g	S.D.	µg/g	S.D.	µg/g	S.D.	µg/g	S.D.	µg/g	S.D.		
Mg	16270	± 2730	13761	± 4025	11830	± 1200	46019	± 5506	46648	± 11969	36571	± 6463		
Si	152332	± 20231	150673	± 20774	152250	± 20475	198640	± 27085	170473	± 23065	187738	± 4966		
Ca	191451	± 6107	205918	± 6551	194399	± 6193	259365	± 8229	224959	± 7156	230146	± 14		
Sc	204	± 12	230	± 16	260	± 13	288	± 15	209	± 13	215	± 21		
Ti	28655	± 2763	29097	± 3562	141	± 15	16910	± 2233	12990	± 1373	13316	± 1462		
V	167	± 11	179	± 14	193	± 10	328	± 19	249	± 17	442	± 27		
Cr	5.43	± 2.54	b.d.l		b.d.l		b.d.l		b.d.l		b.d.l			
Co	158	± 10	135	± 8	144	± 8	135	± 9	115	± 6	87.5	± 2.8		
Ni	198	± 32	179	± 67	102	± 17	65.0	± 13.7	73.6	± 24.6	82.0	± 15.3		
Cu	68.2	± 4.4	48.6	± 3.8	55.1	± 3.9	27.4	± 2.3	174	± 12	64.6	± 2.9		
Zn	5.63	± 1.35	3.85	± 0.97	3.03	± 1.12	2.59	± 1.18	3.53	± 0.83	3.26	± 0.21		
Ga	137	± 7	105	± 7	139	± 8	141	± 9	133	± 8	154	± 2		
Ge	3.14	± 0.79	2.34	± 0.68	2.26	± 0.76	9.62	± 0.83	6.89	± 0.85	18.8	± 0.6		
Rb	0.48	± 0.10	0.36	± 0.07	0.39	± 0.07	0.45	± 0.06	0.43	± 0.07	0.62	± 0.02		
Sr	179	± 8	193	± 10	211	± 10	354	± 19	288	± 13	391	± 13		
Y	132	± 7	155	± 12	160	± 7	275	± 14	204	± 14	285	± 9		
Zr	125	± 6	127	± 10	142	± 6	206	± 9	152	± 10	166	± 7		
Nb	90.3	± 4.1	99.1	± 5.8	110	± 4	199	± 8	151	± 8	247	± 9		
Rh	0.71	± 0.11	0.29	± 0.07	0.16	± 0.03	0.28	± 0.04	0.47	± 0.08	0.90	± 0.26		
Cs	b.d.l		b.d.l		b.d.l		b.d.l		0.05	± 0.03	b.d.l			
Ba	97.3	± 6.5	114	± 8	124	± 7	302	± 19	235	± 15	468	± 32		
La	96.0	± 3.9	69.5	± 3.9	117	± 4	191	± 7	147	± 7	238	± 8		
Ce	163	± 7	131	± 7	182	± 7	264	± 10	204	± 10	323	± 12		
Pr	103	± 5	86.1	± 4.6	117	± 4	151	± 6	116	± 6	173	± 6		
Nd	104	± 5	93.2	± 6.7	120	± 5	191	± 9	145	± 9	211	± 7		
Sm	132	± 7	137	± 12	154	± 8	258	± 15	195	± 15	269	± 9		
Eu	99.7	± 5.1	108	± 7	113	± 4	151	± 7	116	± 7	158	± 5		
Gd	120	± 9	133	± 11	141	± 8	233	± 15	175	± 13	237	± 11		
Tb	117	± 6	139	± 11	142	± 6	221	± 11	168	± 12	229	± 7		
Dy	93.7	± 5.9	113	± 12	116	± 5	139	± 7	106	± 10	144	± 6		
Ho	126	± 7	152	± 15	158	± 7	245	± 12	185	± 16	254	± 9		
Er	71.1	± 4.0	86.8	± 9.6	91.0	± 4.1	247	± 13	188	± 18	254	± 9		
Tm	69.4	± 3.7	85.6	± 8.3	90.3	± 3.4	238	± 10	178	± 15	245	± 8		
Yb	124	± 8	156	± 14	165	± 7	246	± 11	184	± 15	250	± 10		
Lu	126	± 7	154	± 14	162	± 7	229	± 11	172	± 13	227	± 8		
Hf	81.2	± 5.1	95.1	± 8.5	111	± 4	174	± 8	126	± 10	127	± 10		
Ta	102	± 6	115	± 9	133	± 5	144	± 7	109	± 8	152	± 5		
W	20.2	± 1.3	27.0	± 2.6	198	± 8	225	± 11	93.5	± 7.9	146	± 10		
Pb	0.11	± 0.04	b.d.l		0.07	± 0.02	b.d.l		0.09	± 0.02	0.08	± 0.01		
Th	77.9	± 4.0	87.3	± 7.6	103	± 4	233	± 9	177	± 13	296	± 14		
U	0.57	± 0.05	0.45	± 0.05	0.49	± 0.03	11.4	± 0.5	8.18	± 0.70	22.0	± 1.3

**Table 3:** Mineral-melt partition coefficients including the available literature data. The 1σ represents the mean absolute standard error on the average and “n” stands for the number of analyzes that had been incorporated in the calculations for the D-values in the form of “n” of the mineral vs. “n” of the silicate melt.

	**Hibonite**														
	H1-Ti2-R3			H1-Ti5-R4			H1-Ti5-R5			H2-Ti2-R2			H2-Ti2-R3		
	D-Value	σ	n	D-Value	σ	n	D-Value	σ	n	D-Value	σ	n	D-Value	σ	n
Mg	1.86	±0.21	5/6	2.68	±0.22	6/6	2.98	±0.40	6/6	0.92	±0.11	6/6	1.12	±0.12	6/6
Si	0.045	±0.004	5/6	0.027	±0.003	5/6	0.031	±0.003	6/6	0.033	±0.003	6/6	0.051	±0.004	6/6
Ca	0.29	±0.01	5/6	0.31	±0.01	6/6	0.28	±0.01	6/6	0.34	±0.01	6/6	0.32	±0.01	6/6
Sc	0.18	±0.01	5/6	0.19	±0.01	6/6	0.17	±0.01	6/6	0.20	±0.01	6/6	0.18	±0.01	6/6
Ti	0.91	±0.06	5/6	0.68	±0.03	6/6	0.67	±0.05	6/6	1.08	±0.07	6/6	1.21	±0.05	6/6
V	0.033	±0.002	5/6	0.020	±0.001	6/6	0.019	±0.001	6/6	0.044	±0.002	6/6	0.049	±0.002	6/6
Cr	—		3/0	5.24	±2.30	2/1	—		1/0	—		3/0	2.26	±0.58	3/1
Co	1.63	±0.05	5/6	2.40	±0.08	6/6	2.30	±0.10	6/6	0.88	±0.03	6/6	1.13	±0.03	6/6
Ni	1.15	±0.18	5/6	1.91	±0.16	6/6	1.65	±0.23	6/6	0.62	±0.11	6/6	0.77	±0.12	6/6
Cu	0.16	±0.01	5/6	0.18	±0.01	6/6	0.18	±0.01	6/6	0.069	±0.003	6/6	0.12	±0.01	6/6
Zn	3.37	±0.55	4/5	3.53	±0.63	6/6	4.32	±1.07	2/5	2.21	±0.37	4/6	2.44	±0.35	5/6
Ga	2.56	±0.08	5/6	1.81	±0.05	6/6	1.95	±0.07	6/6	1.66	±0.05	6/6	2.44	±0.07	6/6
Ge	2.99	±0.74	3/3	2.80	±0.56	4/6	3.08	±0.83	3/4	3.76	±0.84	5/3	3.89	±0.92	6/3
Rb	—		0/6	—		0/6	—		0/6	—		0/6	—		0/6
Sr	0.49	±0.01	5/6	0.55	±0.01	6/6	0.52	±0.02	5/6	0.52	±0.01	6/6	0.51	±0.01	6/6
Y	0.23	±0.01	5/6	0.17	±0.01	6/6	0.16	±0.01	6/6	0.36	±0.01	6/6	0.31	±0.01	6/6
Zr	0.20	±0.01	5/6	0.19	±0.01	6/6	0.16	±0.01	6/6	0.28	±0.01	6/6	0.25	±0.01	6/6
Nb	0.087	±0.003	5/6	0.083	±0.003	6/6	0.075	±0.003	6/6	0.20	±0.01	6/6	0.19	±0.01	6/6
Rh	19.4	±1.5	5/6	13.6	±1.1	6/6	19.8	±2.5	6/6	—		0/6	34.6	±3.5	6/6
Cs	—		0/0	4.02	±2.39	1/1	—		1/0	—		0/1	—		1/0
Ba	0.031	±0.003	4/6	0.027	±0.003	6/6	0.029	±0.003	5/6	0.025	±0.004	4/6	0.026	±0.003	6/6
La	7.52	±0.19	5/6	4.29	±0.09	6/6	5.17	±0.15	6/6	7.07	±0.17	6/6	8.44	±0.20	6/6
Ce	4.49	±0.11	5/6	2.89	±0.07	6/6	3.05	±0.10	6/6	5.18	±0.13	6/6	5.82	±0.14	6/6
Pr	4.68	±0.12	5/6	2.94	±0.08	6/6	3.23	±0.12	6/6	5.29	±0.13	6/6	5.69	±0.14	6/6
Nd	3.97	±0.12	5/6	2.52	±0.07	6/6	2.69	±0.10	6/6	4.60	±0.14	6/6	4.75	±0.14	6/6
Sm	1.96	±0.07	5/6	1.36	±0.04	6/6	1.34	±0.06	6/6	2.56	±0.09	6/6	2.43	±0.08	6/6
Eu	1.35	±0.04	5/6	0.95	±0.03	6/6	0.93	±0.04	6/6	1.83	±0.05	6/6	1.66	±0.05	6/6
Gd	1.11	±0.04	5/6	0.84	±0.03	6/6	0.81	±0.05	6/6	1.58	±0.05	6/6	1.36	±0.04	6/6
Tb	0.65	±0.02	5/6	0.50	±0.01	6/6	0.48	±0.02	6/6	1.00	±0.03	6/6	0.83	±0.03	6/6
Dy	0.43	±0.02	5/6	0.32	±0.01	6/6	0.31	±0.02	6/6	0.67	±0.03	6/6	0.55	±0.02	6/6
Ho	0.28	±0.01	5/6	0.21	±0.01	6/6	0.20	±0.01	6/6	0.45	±0.02	6/6	0.38	±0.01	6/6
Er	0.18	±0.01	5/6	0.15	±0.01	6/6	0.13	±0.01	6/6	0.30	±0.01	6/6	0.25	±0.01	6/6
Tm	0.11	±0.00	5/6	0.075	±0.002	6/6	0.077	±0.003	6/6	0.19	±0.01	6/6	0.15	±0.01	6/6
Yb	0.069	±0.003	5/6	0.052	±0.002	6/6	0.050	±0.003	6/6	0.12	±0.01	6/6	0.098	±0.004	6/6
Lu	0.053	±0.002	5/6	0.036	±0.001	6/6	0.035	±0.002	6/6	0.088	±0.003	6/6	0.072	±0.003	6/6
Hf	0.49	±0.02	5/6	0.47	±0.02	6/6	0.42	±0.02	6/6	0.66	±0.02	6/6	0.57	±0.02	6/6
Ta	0.35	±0.01	5/6	0.35	±0.01	6/6	0.33	±0.01	6/6	0.74	±0.02	6/6	0.70	±0.02	6/6
W	0.012	±0.004	5/6	0.007	±0.003	4/6	0.009	±0.002	4/6	0.053	±0.013	4/6	0.042	±0.008	6/6
Pb	—		0/4	—		0/3	—		1/0	—		0/6	—		0/2
Th	1.56	±0.05	5/6	0.86	±0.02	6/6	0.81	±0.03	6/6	2.30	±0.08	6/6	2.01	±0.07	6/6
U	—		0/6	0.021	±0.015	1/6	—		0/6	0.038	±0.010	3/6	0.047	±0.018	3/6


	**Hibonite**														
	H2-Ti5-R4			H2-Ti5-R5			H3-Ti5-R4			H3-Ti5-R5			Ø Hibonite		
	D-Value	σ	n	D-Value	σ	n	D-Value	σ	n	D-Value	σ	n	D-Value	σ	
Mg	1.47	±0.13	6/6	1.63	±0.26	6/6	1.04	±0.10	6/6	1.52	±0.25	6/6	1.69	±0.57	
Si	0.032	±0.003	6/6	0.028	±0.002	6/6	0.11	±0.01	6/6	0.050	±0.004	6/6	0.045	±0.011	
Ca	0.35	±0.01	6/6	0.33	±0.01	6/6	0.31	±0.01	6/6	0.29	±0.01	6/6	0.31	±0.02	
Sc	0.17	±0.01	6/6	0.17	±0.01	6/6	0.22	±0.01	6/6	0.23	±0.01	6/6	0.19	±0.02	
Ti	0.87	±0.04	6/6	0.91	±0.06	6/6	0.74	±0.04	6/6	1.01	±0.07	6/6	0.90	±0.15	
V	0.039	±0.002	6/6	0.028	±0.002	6/6	0.098	±0.004	6/6	0.040	±0.002	6/6	0.041	±0.006	
Cr	—		1/0	—		1/0	4.59	±2.21	5/1	—		4/0	4.03	±2.82	
Co	1.46	±0.05	6/6	1.61	±0.05	6/6	0.90	±0.03	6/6	1.45	±0.05	6/6	1.53	±0.14	
Ni	1.26	±0.11	6/6	1.33	±0.27	6/6	0.73	±0.07	6/6	1.15	±0.25	6/6	1.18	±0.51	
Cu	0.13	±0.01	6/6	0.14	±0.01	6/6	0.15	±0.01	6/6	0.12	±0.01	6/6	0.14	±0.02	
Zn	3.73	±1.01	2/5	2.68	±0.41	5/6	1.49	±0.26	5/6	2.27	±0.45	5/5	2.89	±1.51	
Ga	2.18	±0.06	6/6	2.21	±0.09	6/6	1.72	±0.05	6/6	2.33	±0.09	6/6	2.10	±0.19	
Ge	3.57	±0.61	4/6	4.56	±1.00	3/4	2.00	±0.36	4/5	3.82	±0.78	3/5	3.39	±2.02	
Rb	0.86	±0.29	1/6	0.81	±0.25	1/6	—		0/6	—		0/6	0.83	±0.38	
Sr	0.57	±0.02	6/6	0.56	±0.02	6/6	0.48	±0.01	6/6	0.55	±0.02	6/6	0.53	±0.04	
Y	0.23	±0.01	6/6	0.19	±0.01	6/6	0.22	±0.01	6/6	0.17	±0.01	6/6	0.23	±0.02	
Zr	0.18	±0.01	6/6	0.19	±0.01	6/6	0.18	±0.01	6/6	0.27	±0.01	6/6	0.21	±0.02	
Nb	0.11	±0.00	6/6	0.10	±0.00	6/6	0.14	±0.00	6/6	0.22	±0.01	6/6	0.13	±0.01	
Rh	29.2	±2.7	6/6	21.2	±2.2	6/6	8.93	±0.73	6/6	30.0	±3.4	6/6	22.1	±5.5	
Cs	—		0/0	—		0/0	—		0/0	—		0/0	4.02	±2.39	
Ba	0.028	±0.003	5/6	0.028	±0.003	6/6	0.095	±0.005	6/6	0.043	±0.004	5/6	0.037	±0.012	
La	5.76	±0.13	6/6	6.91	±0.21	6/6	3.33	±0.08	6/6	4.83	±0.16	6/6	5.92	±0.43	
Ce	4.16	±0.10	6/6	4.47	±0.13	6/6	2.20	±0.06	6/6	2.90	±0.09	6/6	3.91	±0.29	
Pr	4.08	±0.11	6/6	4.49	±0.13	6/6	2.37	±0.07	6/6	3.12	±0.10	6/6	3.99	±0.31	
Nd	3.42	±0.10	6/6	3.70	±0.14	6/6	2.06	±0.06	6/6	2.54	±0.11	6/6	3.36	±0.31	
Sm	1.86	±0.06	6/6	1.82	±0.09	6/6	1.20	±0.04	6/6	1.32	±0.07	6/6	1.76	±0.19	
Eu	1.33	±0.04	6/6	1.24	±0.05	6/6	0.87	±0.03	6/6	0.93	±0.04	6/6	1.23	±0.11	
Gd	1.10	±0.04	6/6	1.03	±0.05	6/6	0.73	±0.03	6/6	0.78	±0.04	6/6	1.04	±0.12	
Tb	0.67	±0.02	6/6	0.60	±0.03	6/6	0.48	±0.02	6/6	0.46	±0.02	6/6	0.63	±0.06	
Dy	0.44	±0.02	6/6	0.37	±0.02	6/6	0.34	±0.01	6/6	0.31	±0.02	6/6	0.42	±0.05	
Ho	0.30	±0.01	6/6	0.24	±0.01	6/6	0.25	±0.01	6/6	0.22	±0.01	6/6	0.28	±0.03	
Er	0.18	±0.01	6/6	0.16	±0.01	6/6	0.19	±0.01	6/6	0.15	±0.01	6/6	0.19	±0.02	
Tm	0.11	±0.00	6/6	0.089	±0.005	6/6	0.14	±0.00	6/6	0.097	±0.006	6/6	0.11	±0.01	
Yb	0.070	±0.003	6/6	0.058	±0.003	6/6	0.11	±0.00	6/6	0.069	±0.004	6/6	0.077	±0.011	
Lu	0.048	±0.002	6/6	0.040	±0.002	6/6	0.098	±0.003	6/6	0.056	±0.003	6/6	0.058	±0.007	
Hf	0.46	±0.02	6/6	0.46	±0.02	6/6	0.40	±0.02	6/6	0.56	±0.03	6/6	0.50	±0.06	
Ta	0.43	±0.01	6/6	0.46	±0.02	6/6	0.35	±0.01	6/6	0.61	±0.03	6/6	0.48	±0.05	
W	0.017	±0.007	3/6	0.035	±0.007	4/6	0.080	±0.005	6/6	0.058	±0.005	6/6	0.035	±0.026	
Pb	—		0/1	—		0/3	—		0/1	—		0/0	—		
Th	1.38	±0.04	6/6	1.29	±0.06	6/6	0.88	±0.03	6/6	0.85	±0.04	6/6	1.33	±0.14	
U	0.027	±0.013	3/6	0.018	±0.013	2/6	0.092	±0.015	4/6	0.066	±0.018	3/6	0.044	±0.055	


	**Hibonite**			**Melilite**^1^											
	Kennedy et al. 1994			H1-Ti2-R3			H3-R8			Ø Melilite			Beckett & Stolper 1994		
	D-Value	σ		D-Value	σ	n	D-Value	σ	n	D-Value	σ		D-Value	σ	
Mg	0.50			7.78	±1.20	2/6	0.21	±0.01	6/5	3.99	±0.66		—		
Si	0.028			0.70	±0.08	2/6	0.68	±0.06	6/5	0.69	±0.09		—		
Ca	0.30			1.42	±0.05	2/6	1.50	±0.03	6/5	1.46	±0.05		—		
Sc	0.46			1.12	±0.07	2/6	0.012	±0.001	6/5	0.57	±0.04		0.016	±0.007	
Ti	1.29			4.60	±0.29	2/6	0.027	±0.010	1/5	2.32	±0.84		—		
V	—			0.57	±0.04	2/6	0.002	±0.000	6/5	0.28	±0.03		—		
Cr	—			—		0/0	—		0/0	—			—		
Co	—			6.07	±0.27	2/6	0.13	±0.00	6/5	3.10	±0.17		—		
Ni	—			4.25	±0.98	2/6	0.17	±0.03	4/5	2.21	±0.65		—		
Cu	—			1.32	±0.10	2/6	0.044	±0.003	6/5	0.68	±0.07		—		
Zn	—			17.9	±6.5	1/5	0.48	±0.18	2/5	9.18	±4.81		—		
Ga	—			8.47	±0.39	2/6	0.81	±0.03	6/5	4.64	±0.26		—		
Ge	0.78			—		0/3	1.21	±0.27	4/5	1.21	±0.27		—		
Rb	—			—		0/6	0.36	±0.16	1/5	0.36	±0.16		—		
Sr	0.62			2.24	±0.09	2/6	0.52	±0.01	6/5	1.38	±0.07		—		
Y	—			1.17	±0.06	2/6	0.58	±0.02	6/5	0.87	±0.05		—		
Zr	0.35			1.22	±0.07	2/6	—		0/5	1.22	±0.07		—		
Nb	0.27			1.06	±0.05	2/6	0.0003	±0.0001	2/5	0.53	±0.18		—		
Rh	—			123	±13	2/6	0.32	±0.08	3/5	123	±31		—		
Cs	—			—		1/0	—		1/0	—			—		
Ba	0.030			0.53	±0.06	2/6	0.009	±0.001	6/5	0.27	±0.04		0.059	±0.012	
La	5.50			23.4	±0.8	2/6	0.44	±0.01	6/5	11.9	±0.5		0.16	±0.04	
Ce	4.50			15.1	±0.5	2/6	0.41	±0.01	6/5	7.74	±0.33		0.12	±0.04	
Pr	3.80			15.8	±0.6	2/6	0.66	±0.01	6/5	8.23	±0.35		—		
Nd	3.20			13.5	±0.6	2/6	0.76	±0.02	6/5	7.11	±0.38		—		
Sm	1.65			7.39	±0.38	2/6	0.84	±0.02	6/5	4.11	±0.25		—		
Eu	1.25			5.23	±0.22	2/6	0.82	±0.02	6/5	3.03	±0.15		—		
Gd	1.03			4.12	±0.23	2/6	0.88	±0.03	6/5	2.50	±0.16		—		
Tb	0.62			2.69	±0.12	2/6	0.75	±0.02	6/5	1.72	±0.09		—		
Dy	0.36			1.75	±0.12	2/6	0.67	±0.02	6/5	1.21	±0.09		—		
Ho	0.25			1.47	±0.08	2/6	0.60	±0.02	6/5	1.03	±0.06		—		
Er	0.22			1.12	±0.08	2/6	0.49	±0.01	6/5	0.81	±0.06		—		
Tm	0.13			0.87	±0.05	2/6	0.40	±0.01	6/5	0.64	±0.04		0.078	±0.027	
Yb	0.21			0.67	±0.05	2/6	0.33	±0.01	6/5	0.50	±0.04		—		
Lu	0.075			0.71	±0.04	2/6	0.29	±0.01	6/5	0.50	±0.03		—		
Hf	0.73			2.49	±0.14	2/6	0.0004	±0.0001	3/5	1.24	±0.38		—		
Ta	—			2.74	±0.13	2/6	0.0002	±0.0000	4/5	1.37	±0.28		—		
W	—			0.95	±0.20	2/6	0.0002	±0.0001	3/5	0.47	±0.24		—		
Pb	—			—		0/4	—		0/3	—			—		
Th	0.93			5.82	±0.28	2/6	0.088	±0.002	6/5	2.95	±0.16		—		
U	0.080			0.34	±0.20	2/6	0.004	±0.004	1/5	0.17	±0.20		—		


	**Melilite**^1^						**Spinel**^2^								
	Kuehner et al. 1989			Lundstrom et al. 2006			H2-R8			H3-R8			Mel3-R9		
	D-Value	σ		D-Value	σ		D-Value	σ	n	D-Value	σ	n	D-Value	σ	n
Mg	—			—			11.6	±2.7	4/7	10.6	±0.6	6/5	3.83	±0.24	8/5
Si	—			—			—		0/7	0.028	±0.004	1/5	—		0/5
Ca	—			—			0.008	±0.002	2/7	0.010	±0.002	2/5	0.004	±0.001	3/5
Sc	—			—			0.066	±0.010	4/7	0.13	±0.00	6/5	0.058	±0.002	8/5
Ti	—			—			—		0/7	0.12	±0.01	0/5	0.022	±0.001	8/5
V	—			—			0.005	±0.002	1/7	0.025	±0.002	1/5	0.002	±0.001	2/5
Cr	—			—			—		0/0	—		1/0	—		8/0
Co	—			—			12.3	±1.9	4/7	9.82	±0.37	6/5	3.07	±0.14	8/5
Ni	—			—			34.0	±11.3	4/7	36.4	±3.4	6/5	16.9	±2.0	8/5
Cu	—			—			1.81	±0.35	4/7	1.33	±0.06	6/5	0.29	±0.02	8/5
Zn	—			—			20.9	±7.9	4/6	12.2	±2.4	6/5	3.60	±0.90	8/5
Ga	—			—			2.77	±0.40	4/7	1.85	±0.06	6/5	2.65	±0.09	8/5
Ge	—			—			2.41	±1.20	1/2	—		0/5	0.52	±0.23	1/5
Rb	—			0.013	±0.002		1.20	±0.52	1/7	—		0/5	0.61	±0.33	1/5
Sr	0.93	±0.00		0.68	±0.02		—		0/7	0.024	±0.002	1/5	—		0/5
Y	0.22	±0.00		—			—		0/7	0.029	±0.001	1/5	0.0007	±0.0002	3/5
Zr	0.002	±0.000		0.004	±0.002		—		0/7	0.042	±0.012	1/5	—		0/5
Nb	—			0.003	±0.001		0.0009	±0.0003	2/7	0.011	±0.001	2/5	0.0001	±0.0001	1/5
Rh	—			—			—		0/0	59.1	±5.4	6/5	52.1	±3.8	8/5
Cs	—			0.003	±0.001		—		0/1	—		0/0	—		0/0
Ba	—			0.018	±0.001		0.010	±0.005	1/7	0.029	±0.001	1/5	0.003	±0.001	1/5
La	0.35	±0.00		0.056	±0.006		—		0/7	0.0009	±0.0000	3/5	—		0/5
Ce	—			0.053	±0.002		0.0001	±0.0003	1/7	0.001	±0.000	3/5	0.0001	±0.0001	1/5
Pr	—			—			—		0/7	0.0007	±0.0000	4/5	0.0002	±0.0001	1/5
Nd	—			0.066	±0.013		0.0008	±0.0013	1/7	0.001	±0.000	4/5	0.0007	±0.0003	2/5
Sm	0.38	±0.00		0.072	±0.003		0.002	±0.001	0/7	0.002	±0.000	2/5	0.0002	±0.0003	1/5
Eu	—			0.067	±0.005		—		0/7	0.0006	±0.0000	4/5	0.0002	±0.0002	1/5
Gd	—			—			—		0/7	0.002	±0.000	2/5	0.0004	±0.0004	2/5
Tb	—			—			0.0003	±0.0001	2/7	0.0009	±0.0000	4/5	0.0001	±0.0000	5/5
Dy	—			—			0.0006	±0.0011	1/7	0.0010	±0.0001	3/5	0.0003	±0.0002	3/5
Ho	—			—			0.0002	±0.0002	1/7	0.0006	±0.0000	6/5	0.0002	±0.0000	6/5
Er	—			0.037	±0.005		0.001	±0.001	1/7	0.001	±0.000	4/5	0.0003	±0.0001	7/5
Tm	—			—			0.0008	±0.0004	1/7	0.0007	±0.0000	6/5	0.0003	±0.0000	8/5
Yb	0.13	±0.00		0.019	±0.011		0.0008	±0.0006	2/7	0.0008	±0.0001	4/5	0.0007	±0.0001	6/5
Lu	—			—			0.0006	±0.0001	4/7	0.0008	±0.0000	6/5	0.0006	±0.0001	8/5
Hf	—			0.001	±0.001		0.002	±0.001	2/7	0.001	±0.000	5/5	0.0009	±0.0002	6/5
Ta	—			0.003	±0.001		0.0005	±0.0003	1/7	0.002	±0.000	2/5	0.0002	±0.0001	2/5
W	—			—			0.002	±0.001	1/7	0.002	±0.000	2/5	0.0003	±0.0001	4/5
Pb	—			0.33	±0.21		—		0/0	—		0/3	—		0/0
Th	—			0.002	±0.002		0.0004	±0.0002	1/7	0.009	±0.000	4/5	0.0001	±0.0000	2/5
U	—			0.002	±0.002		—		0/7	0.029	±0.029	1/5	—		0/5


	**Spinel**^2^														
	Mel3-R11-Spinel			Mel3-R12-Spinel			Ø Spinel			Lundstrom et al. 2006					
	D-Value	σ	n	D-Value	σ	n	D-Value	σ	n	D-Value	σ				
Mg	3.99	±0.51	6/6	4.97	±0.71	6/12	7.00	±2.20		—					
Si	0.008	±0.002	1/6	0.005	±0.001	2/12	0.014	±0.005		—					
Ca	—		0/6	0.005	±0.001	2/12	0.007	±0.003		—					
Sc	0.063	±0.005	6/6	0.058	±0.005	6/12	0.075	±0.015		—					
Ti	0.028	±0.002	6/6	0.025	±0.002	6/12	0.048	±0.009		—					
V	0.002	±0.001	1/6	0.001	±0.001	1/12	0.007	±0.006		—					
Cr	—		6/0	—		6/0	—			—					
Co	3.70	±0.26	6/6	4.85	±0.26	6/12	6.74	±1.26		—					
Ni	13.8	±2.3	6/6	12.6	±1.4	6/8	22.7	±9.5		—					
Cu	0.42	±0.03	6/6	0.41	±0.02	6/12	0.85	±0.19		—					
Zn	6.65	±0.91	6/5	4.80	±0.51	6/9	9.65	±5.04		—					
Ga	2.77	±0.20	6/6	2.69	±0.13	6/12	2.55	±0.45		—					
Ge	—		0/6	—		0/12	1.47	±0.97		—					
Rb	1.01	±0.42	1/5	—		0/11	0.94	±0.76		—					
Sr	0.002	±0.001	1/6	—		0/12	0.013	±0.007		—					
Y	0.0005	±0.0003	1/6	0.0002	±0.0001	2/12	0.008	±0.007		—					
Zr	—		0/6	0.0006	±0.0003	3/12	0.022	±0.003		0.001	±0.000				
Nb	—		0/6	0.0001	±0.0001	2/12	0.003	±0.004		0.00004	±0.00001				
Rh	69.3	±7.4	6/6	39.9	±11.8	6/11	55.1	±18.5		—					
Cs	—		0/1	—		1/0	—			—					
Ba	—		0/6	—		0/11	0.014	±0.009		—					
La	—		0/6	—		0/12	0.0009	±0.0000		0.000003	±0.000004				
Ce	0.0002	±0.0001	2/6	0.0001	±0.0001	2/12	0.0003	±0.0008		—					
Pr	0.0005	±0.0001	3/6	0.0001	±0.0001	1/12	0.0004	±0.0005		—					
Nd	0.0007	±0.0006	1/6	0.0004	±0.0003	2/12	0.0007	±0.0015		0.00003	±0.00004				
Sm	0.0008	±0.0004	2/6	0.0003	±0.0001	4/12	0.001	±0.002		—					
Eu	—		0/6	0.0003	±0.0002	2/12	0.0003	±0.0005		—					
Gd	—		0/6	—		0/12	0.001	±0.001		—					
Tb	0.0000	±0.0001	1/6	0.0002	±0.0000	4/12	0.0003	±0.0006		—					
Dy	0.0007	±0.0006	1/6	0.0004	±0.0002	3/12	0.0006	±0.0013		—					
Ho	0.0003	±0.0001	4/6	0.0001	±0.0000	5/12	0.0003	±0.0002		—					
Er	0.0004	±0.0001	5/6	0.0004	±0.0001	3/12	0.0006	±0.0006		0.0003	±0.0001				
Tm	0.0003	±0.0001	3/6	0.0004	±0.0001	3/12	0.0005	±0.0003		—					
Yb	0.0004	±0.0005	1/6	0.0005	±0.0002	3/12	0.0006	±0.0010		—					
Lu	0.0006	±0.0001	6/6	0.0002	±0.0000	6/12	0.0006	±0.0002		—					
Hf	0.0003	±0.0004	2/6	0.0007	±0.0003	4/12	0.001	±0.001		0.001	±0.000				
Ta	0.0001	±0.0003	1/6	0.0001	±0.0001	3/12	0.0005	±0.0019		0.0001	±0.0001				
W	0.0003	±0.0005	1/6	—		0/12	0.0010	±0.0020		—					
Pb	—		0/2	3.47	±1.11	1/4	3.47	±1.11		—					
Th	—		0/6	0.0000	±0.0000	1/12	0.002	±0.003		0.0006	±0.0000				
U	0.001	±0.001	1/6	—		0/12	0.015	±0.021		0.0003	±0.0002				


1 The D-values for melilite are directly influenced by the initial Ti concentration within the starting mixture. As far as two samples are appropriate enough to show, it could be that a higher Ti concentration is enhancing the incorporation possibilities for several elements.

2 The D-values for spinel are influenced by the very different starting compositions in respect to the aluminum and magnesium content between the starting mixtures H2, H3 and Mel3 (cf. Table 5)

**Table 4:** Mineral-mineral partition coefficients with corresponding 1*σ* error as the mean absolute standard error of the average.

	**Hibonite/Melilite**		**Hibonite/Spinel**		**Melilite/Spinel**	
	*D*-Value	*σ*	*D*-Value	*σ*	*D*-Value	*σ*
Mg	0.42	±0.16	0.24	±0.11	0.57	±0.20
Si	0.066	±0.018	3.37	±1.47	51.3	±20.2
Ca	0.21	±0.01	46.6	±21.5	217	±100
Sc	0.34	±0.04	2.54	±0.58	7.56	±1.62
Ti	0.39	±0.15	18.8	±4.7	48.6	±20.0
V	0.14	±0.03	5.91	±4.98	41.0	±34.3
Cr	–		–		–	
Co	0.49	±0.05	0.23	±0.05	0.46	±0.09
Ni	0.53	±0.28	0.052	±0.031	0.097	±0.050
Cu	0.20	±0.04	0.16	±0.04	0.80	±0.20
Zn	0.32	±0.23	0.30	±0.22	0.95	±0.70
Ga	0.45	±0.05	0.82	±0.16	1.82	±0.34
Ge	2.81	±1.79	2.31	±2.06	0.82	±0.58
Rb	2.31	±1.48	0.89	±0.83	0.39	±0.36
Sr	0.38	±0.03	40.1	±21.6	105	±56
Y	0.26	±0.03	29.9	±26.1	116	±101
Zr	0.17	±0.02	9.79	±1.63	56.6	±8.1
Nb	0.25	±0.09	–		–	
Rh	0.18	±0.06	0.40	±0.17	2.23	±0.94
Cs	–		–		–	
Ba	0.14	±0.05	2.60	±1.85	19.2	±12.6
La	0.50	±0.04	6420	±522	12,915	±706
Ce	0.50	±0.04	–		–	
Pr	0.48	±0.04	–		–	
Nd	0.47	±0.05	–		–	
Sm	0.43	±0.05	–		–	
Eu	0.41	±0.04	–		–	
Gd	0.42	±0.05	–		–	
Tb	0.37	±0.04	–		–	
Dy	0.34	±0.05	–		–	
Ho	0.27	±0.04	997	±848	3651	±3086
Er	0.23	±0.04	287	±273	1247	±1178
Tm	0.18	±0.02	222	±135	1238	±740
Yb	0.15	±0.02	–		–	
Lu	0.12	±0.01	103	±37	876	±303
Hf	0.40	±0.13	–		–	
Ta	0.35	±0.08	–		–	
W	0.073	±0.066	–		–	
Pb	–		–		–	
Th	0.45	±0.05	–		–	
U	–		–		–	

## 2 Experimental design, materials, and methods

### 2.1 Starting materials

The starting materials compositions are given in Table 5. Starting materials H1 and H2 are based on the starting materials Hib-1 and Hib-6 of Beckett and Stolper [1], our H3 is based on the HB-1 starting material of Kennedy et al. [2]; our starting materials Mel1, Mel2 and Mel3 are similar to the starting materials used by Kuehner et al. ([3], AK40), Beckett and Stolper ([1], AK80) and Lundstrom et al. (CAI-Glass, [4]). In total six different starting material mixtures were prepared from high purity oxides and carbonates. The resulting mixtures were homogenized in an agate mortar under acetone and were subsequently fused in a large Pt-crucible at 1500 °C for at least 3 h in a Linn VMK (Linn Gmbh, Eschenfelden, Germany) high temperature box furnace. The resulting silicate glasses were reground using the same agate mortar with acetone and the resulting powders were doped with 200 µg/g each of Sc, V, Cr, Co, Ni, Cu, Zn, Ga, Ge, Rb, Sr, Y, Zr, Nb, Rh, Cs, Ba, La, Ce, Pr, Nd, Sm, Eu, Gd, Tb, Dy, Ho, Er, Tm, Yb, Lu, Hf, Ta, W, Pb, Th and U, using ICP-MS standard solutions (1000 µg/ml, Alfa Aesar, Germany). However, Ti was added to the hibonite starting mixtures (H1-Ti2, H1-Ti5, H2-Ti2, H2-Ti5 and H3-Ti2, H3-Ti5) using high purity TiO_2_ (Alfa Aesar, Germany).

**Table 5:** Compositions of the starting materials.

	SiO_2_	MgO	Al_2_O_3_	CaO	TiO_2_	MnCO_3_	GeO_2_	K_2_CO_3_
Material	[wt%]	[wt%]	[wt%]	[wt%]	[wt%]	[wt%]	[wt%]	[wt%]
H1	28.2	0.86	42.9	27.3	–	0.17	0.42	0.15
H1-Ti2	27.7	0.84	42.0	26.8	1.99	0.17	0.41	0.15
H1-Ti5	26.8	0.82	40.7	25.9	4.99	0.16	0.40	0.14
H2	31.9	1.89	41.3	25.1	–	0.17	0.30	0.25
H2-Ti2	31.3	1.85	40.5	24.6	1.95	0.17	0.29	0.25
H2-Ti5	30.4	1.80	39.2	23.9	4.90	0.16	0.29	0.24
H3	29.5	2.11	39.7	27.8	–	0.25	0.40	0.30
H3-Ti2	28.8	2.06	38.8	27.1	2.23	0.24	0.39	0.29
H3-Ti5	28.0	2.01	37.8	26.4	4.87	0.24	0.38	0.29
Mel1	29.9	6.30	21.9	40.8	–	0.54	0.24	0.35
Mel2	39.6	11.3	7.52	40.1	–	0.72	0.46	0.35
Mel3	32.9	8.88	26.4	29.1	1.62	0.33	0.47	0.29

### 2.2 Experimental techniques

Experiments were conducted in a vertical tube furnaces (Gero GmbH, Neuhausen, Germany) at atmospheric pressure. We used the so-called “wire-loop technique” [5], [6], [7] where small amounts of starting material powder are mixed with an organic glue (UHU Gmbh, Flinke Flasche, Germany) and suspended on a 0.1 mm thick Pt wire. The loops are about 3 mm in diameter each. Using a home-made platinum wire “chandelier”, several samples could be run simultaneously. The samples were placed in the hot zone of the furnace at 800 °C. The temperature paths were designed so that the samples were first heated to temperatures well above the liquidus (i.e. 1550 °C, *T*_max_ in Table 6), the run was left at 1550 °C (*T*_max_ in Table 6) for at least 8–10 h, and then slowly cooled down to the final run temperature (*T*_quench_) to equilibrate crystals with melts. Most experimental runs were performed with a single cooling ramp, whereas some experiments (H1-Ti5-R5, H2-Ti5-R5, H3-Ti5-R5, H2-R8, H3-R8, Mel3-R9; Table 6) were run with a more complex multi step cooling and heating cycle close to the liquidus temperature. In these runs the experiment was first heated to 1550 °C, then cooled to 1350 °C, left for 10–40 h, then heated with 50 °C/h to 1437 or 1450 °C (c.f. Table 6), left for a few hours, and then cooled to the final run temperature (*T*_quench_). This technique was employed by Kennedy et al. [2] to facilitate crystal growth. However, we found no significant difference between runs with a single cooling ramp compared to the complex heating/cooling experiments. Table 6 shows that the total run time of the experiments was between 100 and 300 h. The experiments were quenched in air by rapidly removing them from the furnace. Details of all experimental parameters are given in Table 6.

**Table 6:** Experimental run conditions. All samples were inserted into the furnace at 800 °C and heated to *T*_max_ with the rate of 100 °C/h. For experiments with complex heating cycles the intermediate steps are given as well. The total duration of the experiments also includes the time for reaching *T*_max_ and the time at *T*_quench_.

Sample	Starting Mix	Run	Heating cycles											
			*T*_max_	Time	Cooling rate	*T*_1_	Time	Heating rate	*T*_2_	Time	Cooling rate	*T*_quench_	Total Time	Phases
			[°C]	[h]	[°C/h]	[°C]	[h]	[°C/h]	[°C]	[h]	[°C/h]	[°C]	[h]	
H2-Ti2-R2	H2	R2	1550	8	-	-	-	-	-	-	5	1450	117.0	hib, gl
H1-Ti2-R3	H1	R3	1550	8	-	-	-	-	-	-	1	1350	333.5	hib, mel, gl
H2-Ti2-R3	H2	R3	1550	8	-	-	-	-	-	-	1	1350	333.5	hib, gl
H1-Ti5-R4	H1	R4	1550	8	-	-	-	-	-	-	5	1450	139.5	hib, gl
H2-Ti5-R4	H2	R4	1550	8	-	-	-	-	-	-	5	1450	139.5	hib, gl
H3-Ti5-R4	H3	R4	1550	8	-	-	-	-	-	-	5	1450	139.5	hib, gl
H1-Ti5-R5	H1	R5	1550	8	5	1350	40	50	1437	24	2	1350	305.7	hib, gl
H2-Ti5-R5	H2	R5	1550	8	5	1350	40	50	1437	24	2	1350	305.7	hib, gl
H3-Ti5-R5	H3	R5	1550	8	5	1350	40	50	1437	24	2	1350	305.7	hib, gl
H2-R8	H2	R8	1550	10	5	1350	10	50	1450	10	2	1350	140.0	an, sp, gl
H3-R8	H3	R8	1550	10	5	1350	10	50	1450	10	2	1350	140.0	mel, sp, gl
Mel3-R9	Mel3	R9	1550	10	5	1350	10	50	1450	10	2	1200	142.5	mel, sp, gl
Mel3-R11	Mel3	R11	1550	8	-	-	-	-	-	-	3	1200	193.0	sp, gl
Mel3-R12	Mel3	R12	1550	8	-	-	-	-	-	-	3	1000	211.0	sp, gl

an = anorthite, gl = glass, hib = hibonite, mel = melilite, sp = spinel

The samples were mounted in epoxy resin, polished, and pre-examined using optical microscopy and a JEOL JSM-6610 LV SEM scanning electron microscope equipped with EDX system at the University of Münster. Samples that contained hibonite, melilite or spinel large enough for further chemical characterization were subsequently analyzed for major and trace elements.

### 2.3 Analytical techniques

Major elements analyses were performed with a JXA-8530F Hyperprobe field emission electron beam microprobe analyzer (EMPA) at the University of Münster. Operating at 15 kV acceleration voltage, a beam diameter of 3 μm and 5 nA beam current for the silicate melts and 15 nA for the minerals. We used a five WDX detector setup with two TAP crystals (Mg, Al), two PET (Ca, Si) and one LiF crystal (Ti). Natural and synthetic materials that were used for standardization are: jadeite (Na_2_O), kyanite (Al_2_O_3_), sanidine (K_2_O), Cr-diopside (Cr_2_O_3_), diopside (CaO), San Carlos olivine (MgO), fayalite (FeO), hypersthene (SiO_2_), rhodonite (MnO) and rutile (TiO_2_). A number of secondary standards (chromite, olivine, cr-diopside) were measured as unknowns to monitor external precision and accuracy.

Trace elements were measured by with a ThermoFisher Element II sector field ICP-MS coupled to a Photon Machines AnalyteG2 ArF Excimer laser at the University of Münster, operating with a 4 J/cm^2^ laser fluency and a repetition rate of 5 Hz. A HelEx 2-volume sample cell was used which holds up to 8 one-inch diameter mounts, 6 thin sections and additional reference materials. Prior to sample analyses, the system was tuned with the NIST SRM 612 for high sensitivity, stability, and low oxide rates (^232^Th^16^O/^232^Th < 0.2%). Spot sizes for analysis were between 35 and 50 μm in diameter, while the 50 µm where mainly used for the silicate glasses. Total measurement time was 75 s with 40 s ablation time on the sample and 20 s on the background, the wash out delay was 15 s.

The NIST 612 standard glass [8] was used as an external standard and the BIR-1G [8] and BCR-2G [8] were analyzed as unknowns over the course of this study to monitor precision and accuracy. Twelve sample measurements were bracketed by three measurements of the NIST 612 glasses. For the hibonite and melilite crystals, ^43^Ca was used as an internal standard, for spinel ^26^Mg and for the silicate melts ^29^Si was used internal standard element.

## Appendix A Supplementary material

Supplementary Table A1.

Supplementary Table A2.
